# Stability assessment of surrounding rock in downward mining route supported by slab-wall backfill structure

**DOI:** 10.1038/s41598-024-64620-5

**Published:** 2024-06-14

**Authors:** Yu Yin, Shijiao Yang, Yan He, Jian Pan, Zhenpeng Guo, Junwei Fan, Zhipeng Wang

**Affiliations:** 1 School of Resources Environment and Safety Engineering, University of South China, Hengyang, 421001 Hunan People’s Republic of China; 2 Sinosteel Maanshan General Lnstitute of Mining Research Co., Ltd, Maanshan, 243000 Anhui People’s Republic of China; 3 State Key Laboratory of Safety and Health for Metal Mines, Maanshan, 243000 Anhui People’s Republic of China; 4 School of Civil Engineering, University of South China, Hengyang, 421001 Hunan People’s Republic of China

**Keywords:** Backfill mining, Slab-wall structure, Numerical simulation, Ground pressure, Stability analysis, Solid Earth sciences, Risk factors, Engineering

## Abstract

Characteristic of ground pressure in surrounding rock is generally considered as the theoretical basis of parameter optimization for stope structure and technology. To explore the feasibility of efficient method for the second-step downward route backfill stopes in Shanjin gold mine, various numerical simulation methods were used to investigate the effect of slab-wall backfill structure on stability of surrounding rock in downward route mining system. The maximum principal stress, artificial false roof stress, and displacement were analyzed to evaluate the level of ground pressure in different mining areas. These results indicate the optimized structural parameters for backfill stopes, which may also provide a low-cost way to achieve a high safety for downward route mining system.

## Introduction

Downward backfill mining method reveals significant advantages in the mining technology of unstable or high-value ore deposits. However, there are still some urgent challenges on the regarding the engineering application of this technology, such as large labor intensity, high support cost, and poor ventilation condition^1,2^. For the purpose of exploring an economic and environmental way to enhance the sustainability for mining works, computer-aided modeling methods have been widely used in underground mining technology, such as backfill process, ventilation optimization, and stability assessment^3^. This may involve the application of intelligent algorithms in automation and simulation technology, which is possible to enhance the safety and efficiency of mining works^4,5^.

Wang et al. demonstrated that downward backfill mining method can significantly enhance the production capacity of the underground crushing mine, by optimizing the mining scheme of this method^6^. Yao et al. explored the mechanical mechanism of hexagonal mining route by analyzing the stress distribution conditions during the process of smooth blasting and route expansion^7^. Currently, numerical simulation becomes one of the major methods to assess the stability of backfill stopes^8^. Hu et al. revealed the deformation mechanism of the surrounding rock after backfilling, via establishing the relationship between the roof displacement and backfill strength^9^. These findings may provide a theoretical calculation method to determine the strength requirements of the artificial roof in backfill stopes.

Previous researches have obtained some achievements on backfill mining technology by developing new-type backfill material and improving mechanized level. However, some critical issues, such as high cost, low stability, and adverse ventilation, still restrict the large-scale application of downward route backfill method^10,11^. Thus, the present study focuses on the stability of surrounding rock in downward mining route supported by slab-wall backfill structure. The stress distribution and transfer mechanism of the backfill stopes in Shanjin gold mine were investigated using numerical simulation. Results of the present study may provide a feasible way to optimize mining work and enhance the sustainability for underground mines.

## Downward route backfill scheme

Downward route backfill is the main mining method used in Shanjin gold mine, due to its crashed rock mass in the underground mining area. The stage height, sublevel height, and layer height are 40 m, 10–15 m, and 4.0 m, respectively. The structural parameters of backfill stopes are designed as a length and width in accordance with the strike direction and horizontal thickness of the ore body, falling between 40 and 60 m. The joints are mainly located in the center of the ore body, exhibiting a trend of vertical development. The backfill stopes are designed into 10–12 layers for a single stage, with excavation sequence in order from top to bottom. Each layer can be further divided into several mining rooms, with an average length and width of 20–40 m and 4.0 m, respectively. Rock drilling is completed using a YT28 air-leg rock drill. Each stope is excavated for about 7 days under the protection of installed anchor rod, followed by backfilling the goaf for about 2 days. After consolidating for about 14 days, the artificial roof is formed with a compressive strength of 4.0 MPa. The extracted ore are loaded into an electric locomotive through the drawing funnel installed at the bottom of the stope, subsequently transported to the wellhead of the skip and elevated to the ground surface route.

In addition, the backfill stopes are executed according to a two-step route: one-step stope near the top and bottom layers and two-step stope at the middle layers. The backfill recipes for each step are designed as two types: high-strength (generally at the bottom layers) and standard-strength, as to satisfy the requirements of backfill mining. After laying crushed stone cushion with a plastic film, backfill slurry is filled into the stope via the pipeline installed on the wall of the backfill roadway using hooked wire mesh. Subsequently, an artificial overflow channel can be formed by erecting reinforcement mesh and constructing backfill retaining walls. It is also required the compressive strength higher than 4.0 MPa for the high-strength layers with a backfill height of 1.0 m, and higher than 1.0 MPa for the standard-strength layers with a height of 3.0 m. For the purpose of improving efficiency and reducing cost, four comparative schemes were formulated according to the production status of Shanjin gold mine. A novel technology of two-step and non-joint top filling was applied for these schemes, to accelerate the transition to the subsequent mining cycle^12^. Therefore, it is necessary to analyze and assess the stress distribution and transmission, ensuring the stability of the backfill stopes, which may thus provide an important theoretical foundation for on-site mining works route.

**Figure 1:**
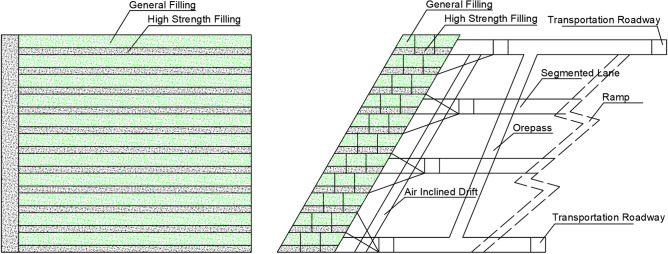
Schematic diagram of the control scheme.

The control scheme (Fig. 1) refers to the traditional joint top backfill scheme, where the bottom layers are filled with high-strength slurry at a cement-tailing ratio of 1:6, and the upper layers are filled with standard-strength slurry at a cement-tailing ratio of 1:20.

Four comparative schemes have been designed as follows.

**Figure 2:**
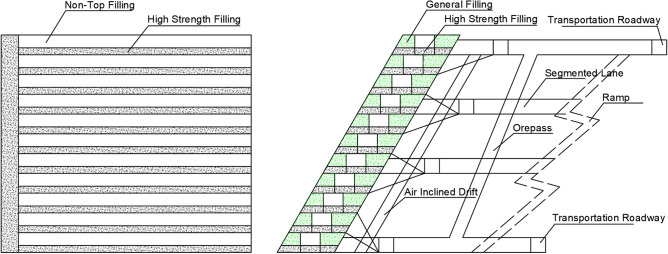
Schematic diagram of full non-top filling scheme (I).

Scheme I: full non-top filling scheme (Fig. 2), the bottom layers are filled with high-strength slurry at a cement-tailing ratio of 1:6, and the upper layers are unfilled and retained after excavation.

**Figure 3:**
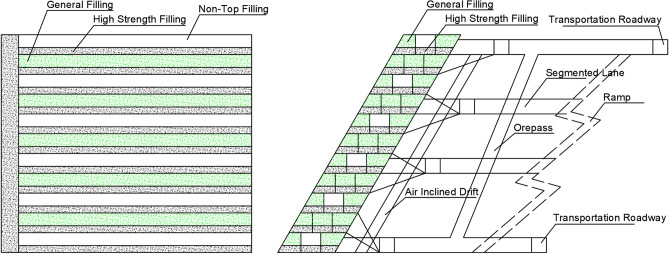
Schematic diagram of filling every other one scheme (II).

Scheme II: filling every other one scheme (Fig. 3), the first layer is filled with high-strength slurry at a cement-tailing ratio of 1:6, followed by the excavation of next layer remaining unfilled.

**Figure 4:**
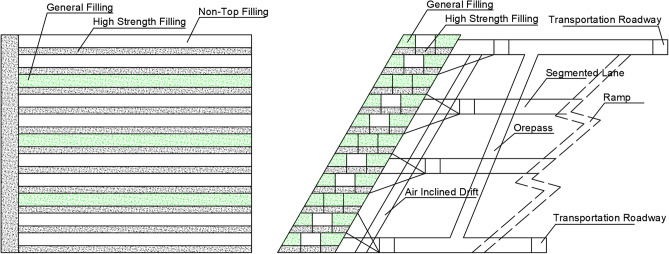
Schematic diagram of filling every other two scheme (III).

Scheme III: filling every other two scheme (Fig. 4), the first layer is filled with high-strength slurry at a cement-tailing ratio of 1:6, followed by the excavation of the second and third layers remaining unfilled.

**Figure 5:**
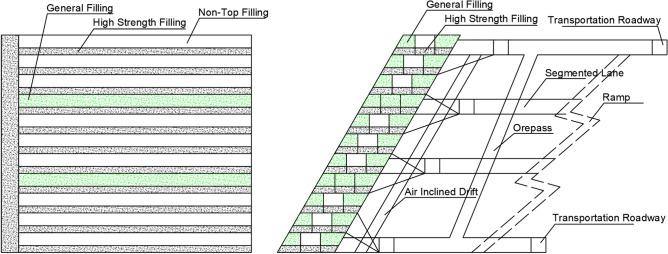
Schematic diagram of filling every other three scheme (IV).

Scheme IV: filling every other three scheme (Fig. 5), the first layer is filled with high-strength slurry at a cement-tailing ratio of 1:6, followed by the excavation of the second, third, and fourth layers remaining unfilled, mainly applied for excavating the low stopes route.

## Numerical simulation study of pressure emergence pattern in mining field

### Rock mechanical parameter

The rock mechanical parameters were determined according to the results of mechanical tests, as shown in Table 1. It is noticeable that the surrounding rock and orebody samples for mechanical tests were cut and prepared from the mining cores.

**Table 1:** Mechanical parameters of surrounding rock, orebody, and backfill samples.

Categories	Density/Mg/m^3^	Tensile strength/MPa	Bulk modulus/GPa	Shear modulus/GPa	Rock Shear Strength	
					c/MPa	φ/°
Surrounding rock	2.65	0.1	4.6	2.8	1.3	45
Orebody	2.65	0.05	2.9	1.7	0.9	40
1:6 high-strength backfill	2.1	0.2	1.3	0.8	0.4	35
1:20 standard-strength backfill	2.1	0.01	0.33	0.2	0.1	25

### Numerical model establishment

**Figure 6:**
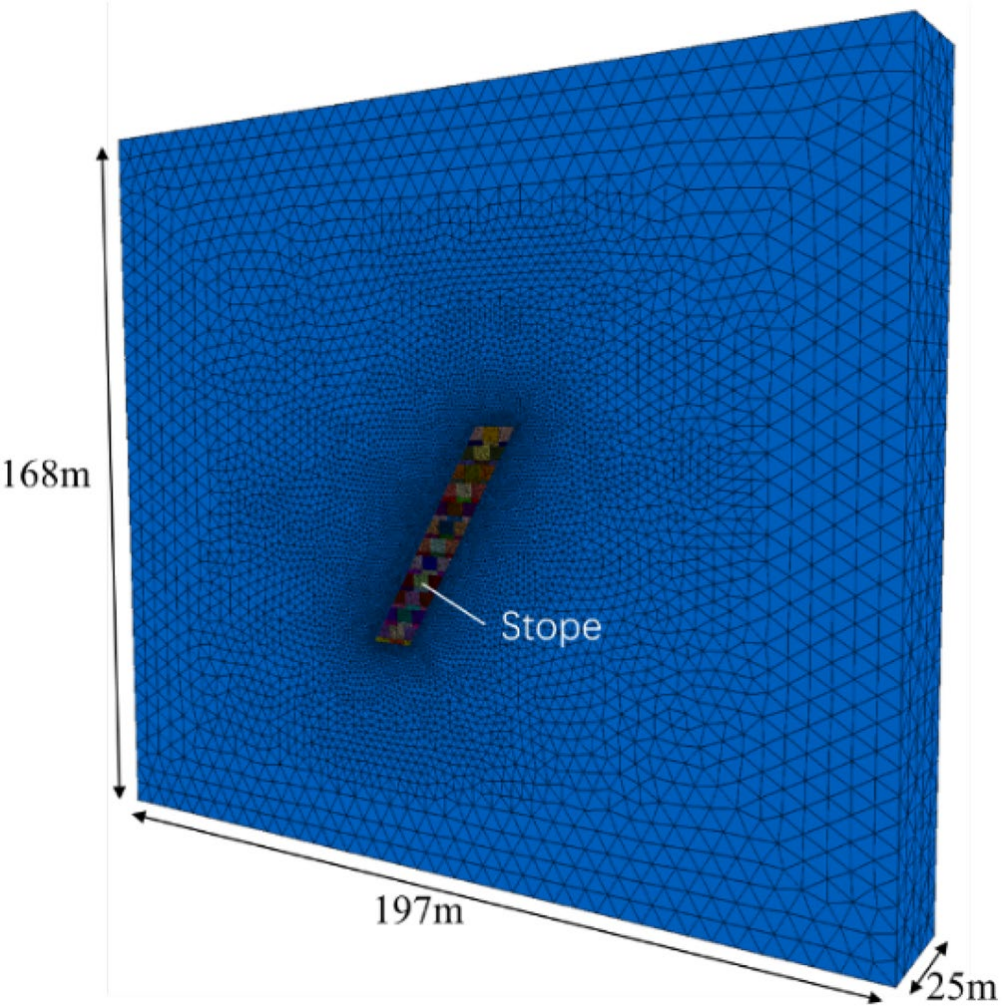
Schematic diagram of numerical model.

Figure 6 shows the three-dimensional numerical model of the backfill stopes established using FLAC3D program, with consideration on stage height and ore body occurrence^13,14^. It should be noted that this model is idealized by appropriately simplifying the boundary condition, due to its complex practical factors. This simplification helps to provide an intuitive understanding of the stress and displacement for surrounding rock, orebody, and backfill. For numerical calculation, Mohr–Coulomb criterion was adopted as the failure criterion, and the initial vertical and horizontal stress values were determined according to the self-weight stress of surrounding rock, orebody, and backfill^15^.

**Figure 7:**
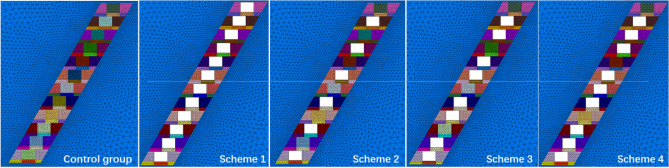
Model sections of different slab wall structure backfill schemes.

Figure 7 displays five model sections at − 20 m stage for the control scheme and designed scheme 1–4, satisfying the requirements of a minimum mining elevation of − 20 m specified by the mining license. As a comparison, both of the control scheme and designed scheme models were further divided into 12 layers with a layer height of 4.0 m. The structural parameters of the backfill stopes were determined to be 197 × 4.0 × 168 m^3^ (length × width × height). In total, these models were divided into 695 thousand tetrahedral grids through 125 thousand nodes for subsequent numerical analysis.

### Analysis of simulation results

#### Initial ground stress

**Figure 8:**
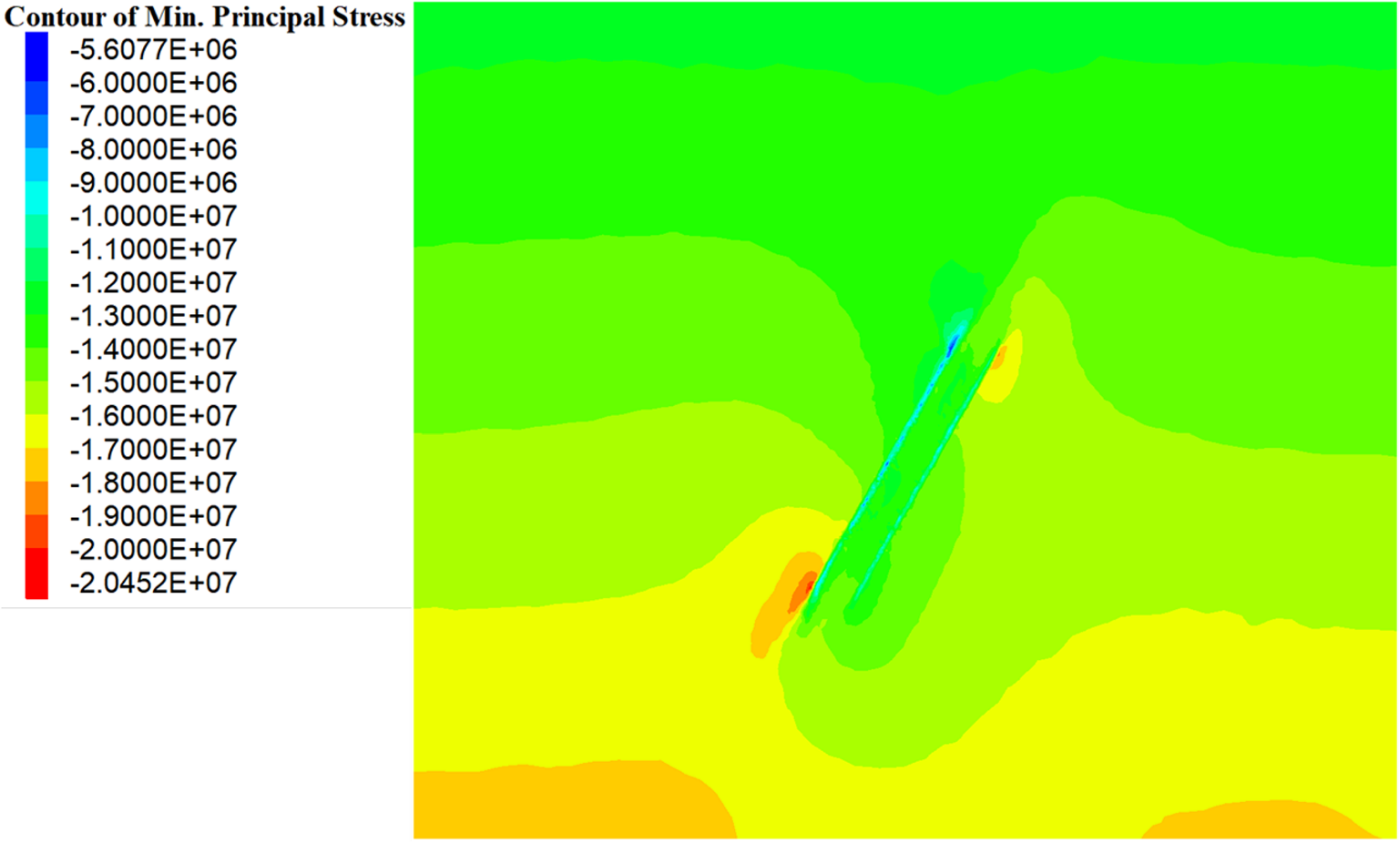
Initial stress equilibrium.

In order to keep the simulated stress consistent with the actual stress, numerical calculation were performed at the beginning of stress equilibrium, as shown in Fig. 8. With considerations of minimum mining elevation (− 20 m), mining depth (500 m), self-weight stress, the results of initial stress calculation demonstrated the maximum principal stress values of 14.0 MPa (middle layer) and 17.5 MPa (bottom layer).

#### Distribution pattern of maximum principal stresses in the stope perimeter rock

After excavation, evident deformation and stress release can be found in the surrounding rock near the stopes, due to the failure of original rock support. Thereafter, the released stress will be transferred to the far surrounding rock through the overlying rock layers. However, the stress can not be sufficiently released from the far surrounding rock, mainly attributed to its limited deformation, contributing to significant stress increases in far surrounding rock^8,16,17^.

**Figure 9:**
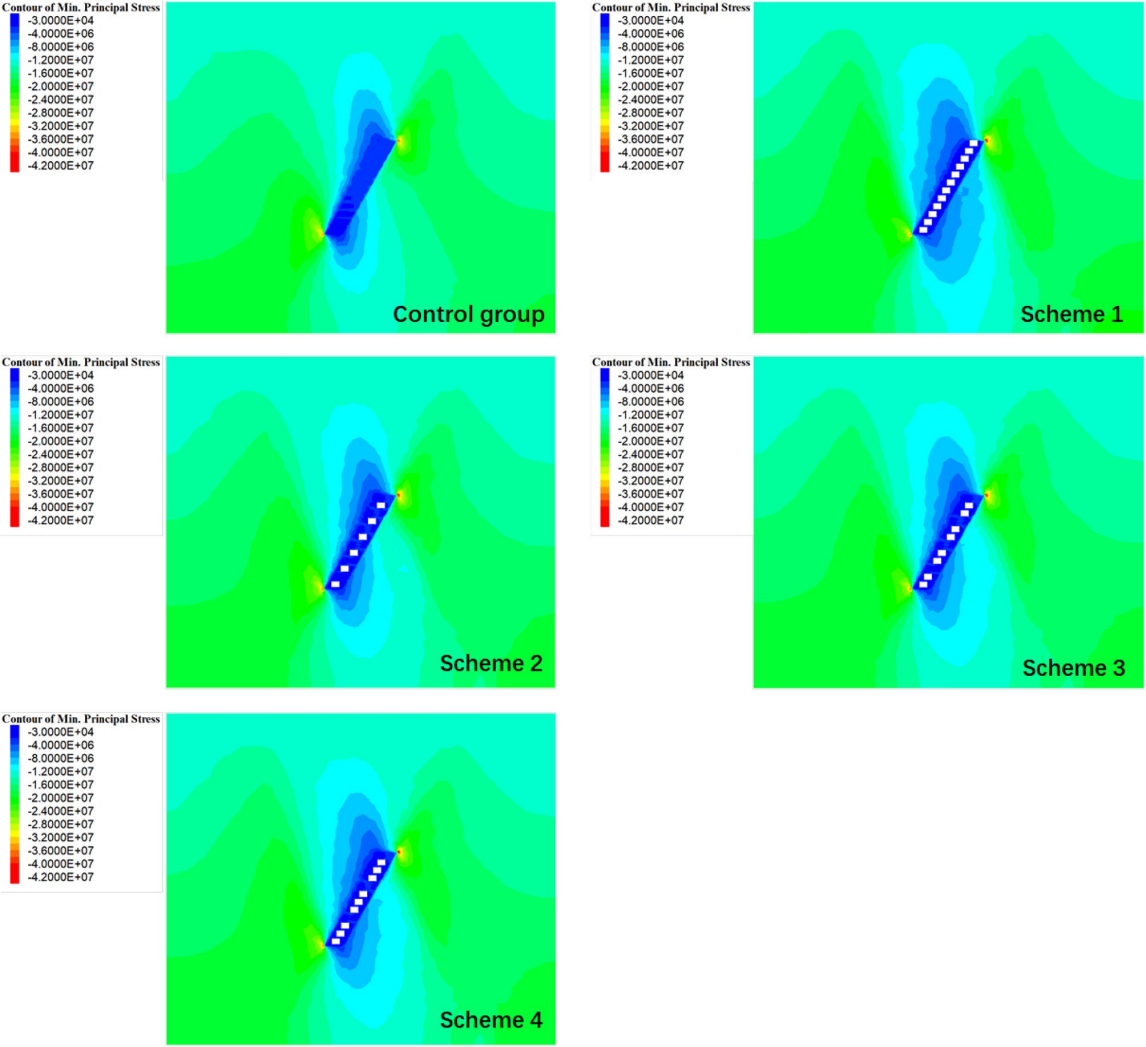
Cloud diagram of the maximum principal stress.

For the purpose of assessing the strength and weakness of each backfill scheme, the stress distribution and mechanics behavior were analyzed by comparisons of maximum principal stress. As seen in Fig. 9, the blue areas represent lower stress regions, and red areas represent higher stress regions. It is evident that, after mining and backfilling, both the surrounding rock and backfill on the upper and lower layers have experienced reduced stress levels, with the upper layer demonstrating lower stress levels and wider reduction areas compared to the lower layer. In addition, Fig. 9 also indicates significant stress increases in the far surrounding rock, with concentration areas pronounced in the upper-right and lower-left corners of the backfill stope.

**Figure 10:**
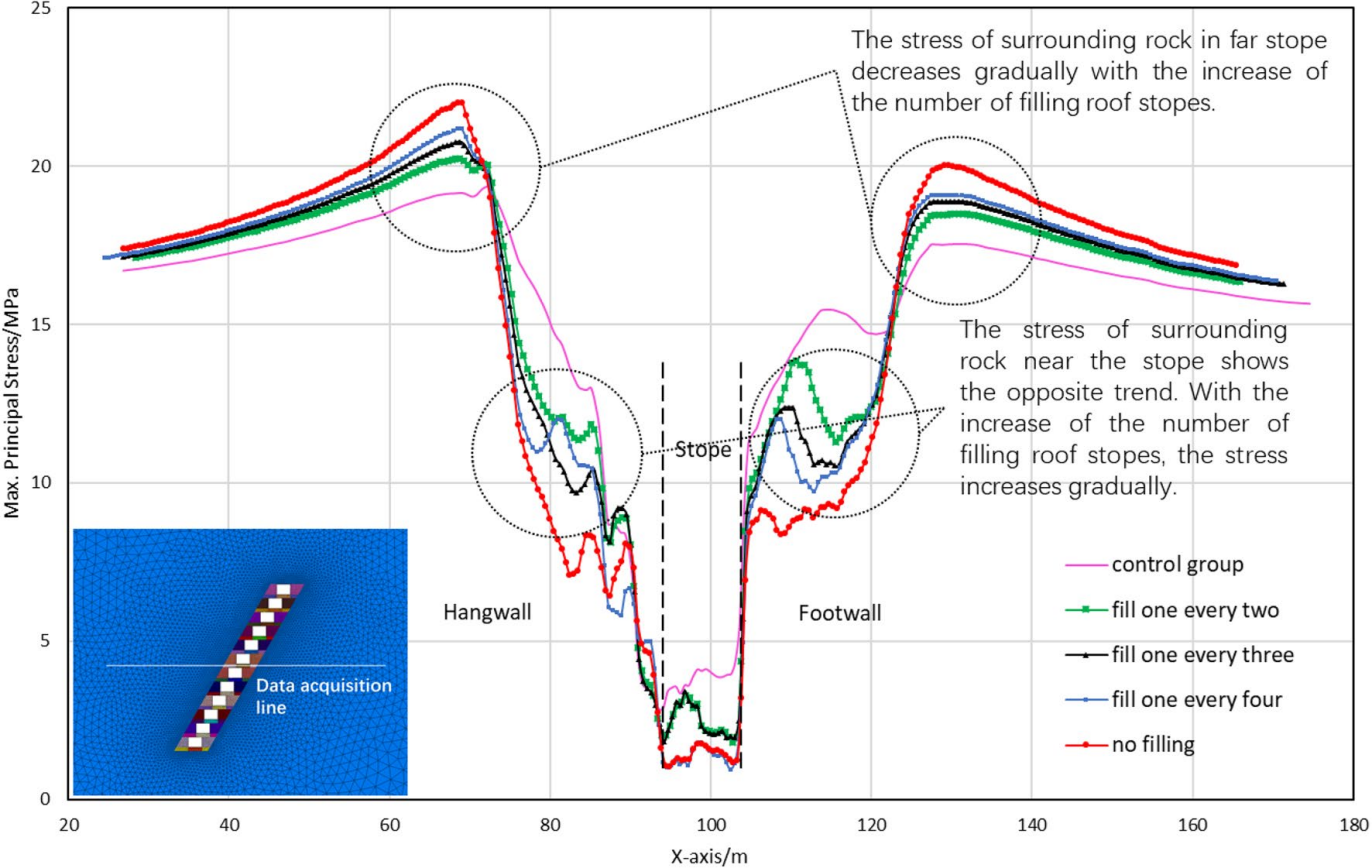
Maximum principal stress variation curve of the backfill and surrounding rock of the stope.

The previous description provides a fundamental overview of stress release and transfer patterns in the surrounding rock. While the alleviated or intensified degree for the stress in surrounding rock may also be influenced considerably by different backfill schemes. Thus, the data related to the stress distribution were collected and analyzed to obtain the stress variation curves for both the surrounding rock and backfill when using different schemes, as shown in Fig. 10.

According to the stress distribution characteristics shown in Fig. 10, it is reasonable to conduct a further discussion on the following regions. It is particularly noticeable that a safety factor margin of 30% has been considered for the calculated stresses, to reduce the impact of various uncertain factors on mining safety.

**Figure 11:**
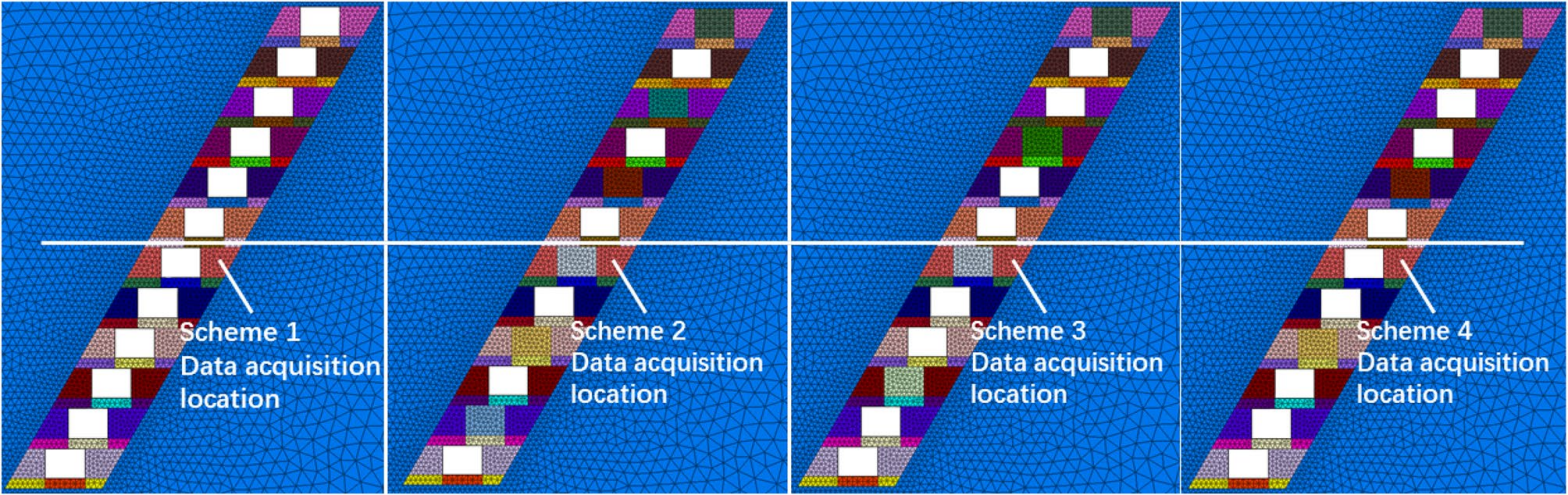
Schematic diagram of the location of the stope and perimeter rock stress collection.

1. Far surrounding rock region: located approximately 20–30 m away from the edge of stopes, exhibiting significant increases in maximum principal stresses ranging from 14.0 to 23.0 MPa for the surrounding rock. It is also observed that the stress values of the far surrounding rock increased constantly with the decrease in numbers of top backfill stopes. This may be explained by the effect of extra stress transferred to the far surrounding rock, due to the reduction of load-bearing capacity for the backfill stopes^18,19^. Engineering practice indicates that the safety risks caused by stress concentration can be effectively mitigated via enhancing the compressive strength of the surrounding rock with consideration of a safety factor margin^20^. Thus, the results of mechanic tests indicate the compressive strength value of 50.0 MPa, far higher than the maximum principal stress in the stress concentration area, which implies a good stability in the far surrounding rock region.
2. Near-stope surrounding rock region: located approximately 15 m away from the edge of stopes, showing gradual decreases in maximum principal stresses for the surrounding rock. The stress level in this region fluctuates with changes in numbers of top backfill stopes, exhibiting an opposite trend compared to those for far surrounding rock region. A higher backfilling rate of the stope roof can be responsible for the increase in load-bearing capacity of the near-stope surrounding rock, which implies that the near-stope rock also shares the load of rock stress^21–23^. The limited deformation, to a certain extent, impedes the stress release and fissure development, contributing to the enhanced integrity and load-bearing capacity for the near-stope surrounding rock^24^. As confirmed by Fig. 10, the maximum principal stress of the near-stope surrounding rock decreased from 7.7 to 3.5 MPa, attributed to the displacement constraint imposed by the backfill body.
3. High-strength backfill region: located at the bottom of the backfill stope, revealing significant differences in the maximum principal stresses among various schemes. The non-top filling scheme I consistently yields the highest maximum stress in the backfill at the stope bottom, followed by the filling every other one II and filling every other two III schemes. The lowest stress level was observed in the filling every other three IV and non-top filling scheme I schemes. As indicated by Fig. 11, it becomes evident that Schemes II and III, featuring high-strength backfills (artificial roofs), share similar conditions characterized by lower stope backfill and roof jointing. In addition, Schemes I and IV illustrate substantial impacts of the degree of top connection for lower stopes on the stress distribution of artificial false roof.

Overall, the backfill stope demonstrates positive effects on stress transfer and release in the surrounding rock. It effectively utilizes backfill capacity to curtail surrounding rock displacement, thereby changing the stress distribution to mitigate the stress concentration around the backfill stopes. It is noteworthy that the load-bearing capacity of the backfill body is limited by the stiffness of the surrounding rock. The lower stiffness of backfill body compared to surrounding rock may be considered as the main reason for the weaker capacity to absorb the transmitted stress^25^. Therefore, both the increases in backfill volume and strength can not maximize the utilization of self-supporting capacity of the surrounding rock^26^. Instead, it may result in substantial costs due to the excessive safety factor margin for the backfill stopes^27^.

To achieve a balance between safety and economic factors, selecting an appropriate backfill strategy requires careful consideration of the mining method and the quality characteristics of the surrounding rock^28^. Prioritizing mine safety allows for optimizing stope backfill volume by leveraging the inherent load-bearing capacity of the surrounding rock and pushing the upper limits of the load-bearing capacity of backfill body. The reduction in backfill volume not only provides cost savings but also enhances the efficiency of stope operations.

#### Comparative analysis of artificial false roof stresses

The mining method utilized in this mine is the downward route backfill, entailing a specific sequence of ore extraction. Initially, ore bodies near the upper and lower layers of the stope are extracted, followed by the intermediate layers. Notably, top backfill connection has already been executed in the upper and lower layers to ensure the stability of surrounding rock. The stability of the intermediate layers depends mainly on the integrity of the artificial false roof in the stope.

**Figure 12:**
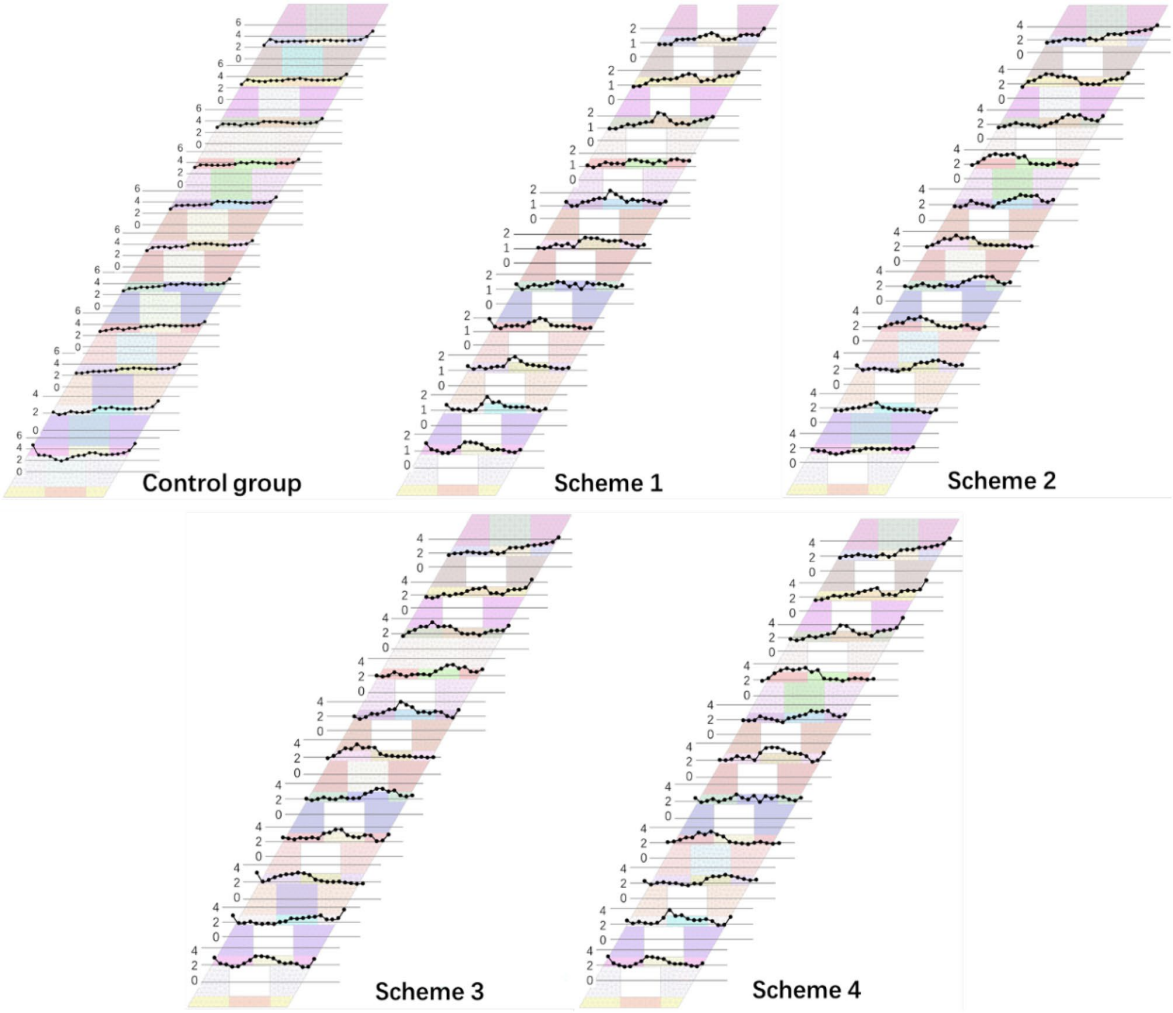
Maximum principal stress curve.

According to the discussion in Sect. “distribution pattern of maximum principal stresses in the stope perimeter rock”, it is evident that the internal stress magnitude in the artificial false roof depends on whether the upper and lower stopes are backfilled. The stability of the artificial false roof significantly influence the safety of the mining operation in the lower stopes^29,30^. To ensure the safety of the artificial false roof, a comparison is performed between the compressive strength of the artificial roof and the maximum compressive stress calculated by numerical simulation. During the calculation, an elastic–plastic model was established to analyze the changes in maximum principal stress with different schemes, with the results shown in Fig. 12.

Referring to the strength data of the artificial roof using backfill method, collected from the typical underground mines around the world, as summarized in Table 2^31–33^. The following discussions on stability assessment of the backfill stopes under different excavation schemes can be reached.

**Table 2:** Strength of artificial roof varies around the world.

Mine	Country	Backfill material	Compressive strength/MPa
Jinchuan nickel mine	China	Gobi sand	5.0
Karatongk copper mine	China	Gobi sand	4.0
Maoping lead–zinc mine	China	Full tailings	4.0
Garpenberg zinc mine	Switzerland	Full tailings	3.0
Orkush lead–zinc mine	Poland	Full tailings	2.5
Penarroye mine	France	Concrete	7.0

1. The maximum principal stresses were maintained below 4.0 MPa in all the schemes, with isolated areas close to the surrounding rocks of the lower plate experiencing maximum principal stresses of up to 5.0 MPa. The stresses are higher when the upper and lower stopes of the artificial roofs are backfilled, decreasing in their absence. It is also noticeable that the calculated internal stresses are lower than the compressive strengths of the artificial roofs during the numerical simulation process.
2. The maximum principal stresses in the artificial roofs correlate with the presence or absence of backfilling in upper and lower layers. Under ideal elastic–plastic conditions, the artificial roofs can be considered in a plastic deformation stage when the stope is not backfilled, resulting in enhanced elastic deformation and stress release, thereby reducing the values of maximum principal stress.
3. The internal stresses in the artificial roof transferred from the upper to the lower level of the stope, exhibiting a trend of gradual decline with the stope excavation. This may be explained by that the lower layers maintain integrity while the upper layers are excavated, contributing to marked increases in stress and displacement in both the surrounding rock and backfill^34–36^. Thus, the lower stresses distributed in the lower layers reveal positive effects on the stability of the backfill stopes, whereas the higher stressed existing in the upper artificial roof decline the stability of the stopes with an extended period.
4. As compared to the full non-top filling scheme I, the interval backfill schemes II-IV improve the stability in the artificial roof, due to the redistribution from one-dimensional stress to two-dimensional and three-dimensional stress exerted by the surrounding rock on both sides.

In summary, the compressive stress value in the artificial roof depends mainly on the stope structure and backfilling scheme^37^. Results of numerical simulation indicate that the internal compressive stresses in the artificial roof remains were maintained lower than the safety threshold of 4.0 MPa. It is particularly noticeable in the case of the internal stress ranging around 2.0 MPa, significantly lower than the compressive strengths of the backfill.

#### Displacement analysis

Stress and displacement are two correlative parameters widely used for analyzing the stability of the surrounding rock. During the stope extraction, the stresses in the original ore body can be transferred into the surrounding rock, resulting in both volumetric and shear deformations for the surrounding rock with the floor acting as the space for deformation. As a result of the rock deformations, the internal stress loads are gradually released and transferred to the far surrounding rock. This transmission continues until the far surrounding rock is constrained by space, ceases further deformation, and no longer transmitted to deeper layers. Therefore, displacement plays a vital role in analyzing the stress transfer characteristics and the integrity of the surrounding rock.

**Figure 13:**
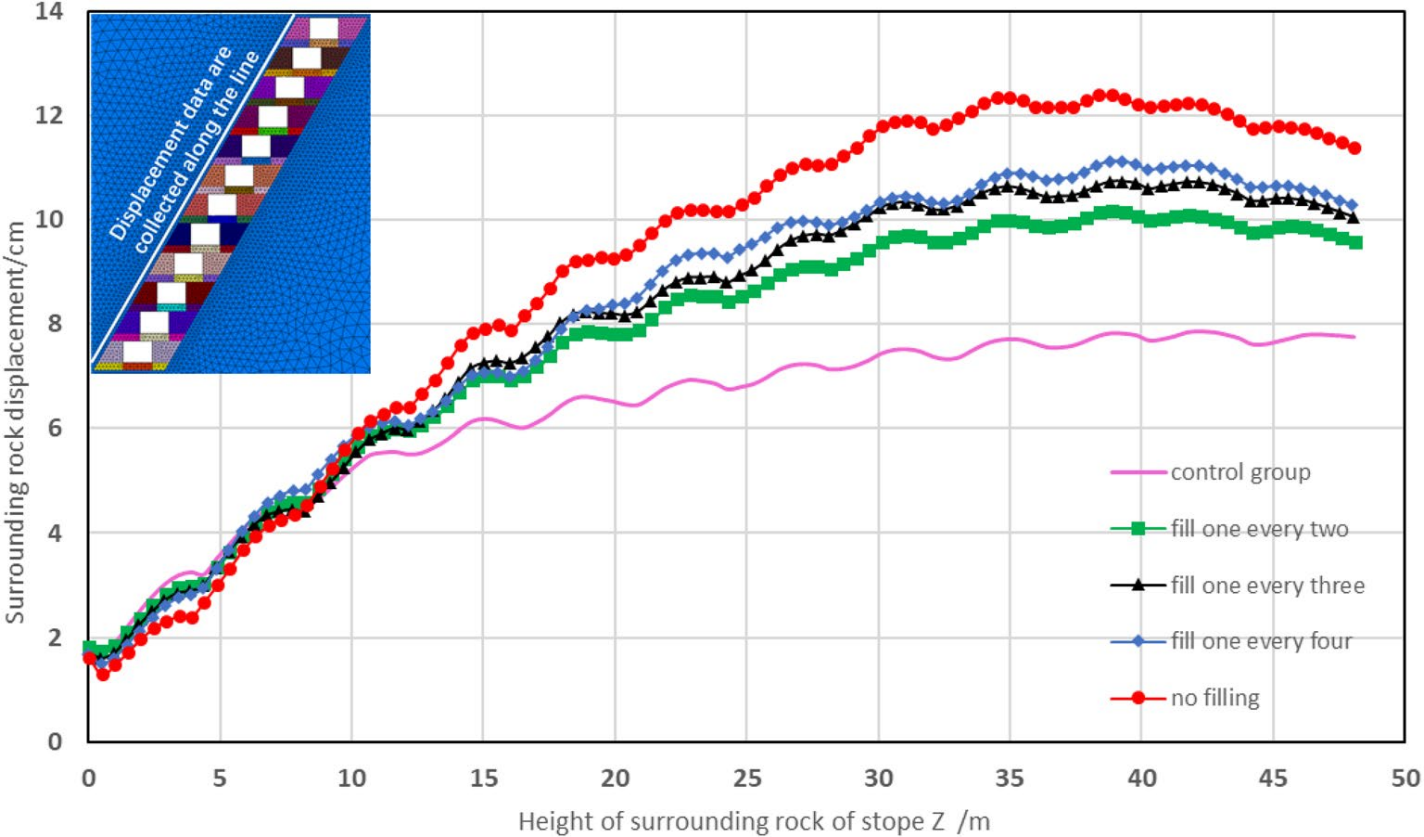
Displacement curves of the surrounding rock.

Vertical displacement has been considered as the major contributor to the rock displacement, which is more pronounced while the upper layers are excavated. The displacement of the surrounding rock near upper layers significantly increased with the increase in numbers of the stopes without top connecting. The displacement curves were than generated by collecting the displacement data of upper surrounding rock under different schemes, as shown in Fig. 13.

1. Two significant increases in displacement values were observed for the surrounding rock in the upper layers of the backfill stopes under different schemes. The first increase occurred during the process of full top-connection and partial top-connection, while the second was found in the partial top-connection and no top-connection schemes. This may reveal a substantial effect of stope backfilling on reducing the displacement of surrounding rock in the upper layers.
2. The displacement of surrounding rock gradually increases in the vertical direction away from the working area, reaching the maximum near the top of the middle layers. Significant differences in the displacement of surrounding rock under different filling schemes were also observed away from the bottom working area.
3. Within a range of 10–12 m from the bottom of working area in this mining stage, there is no evident change in the displacement of the upper surrounding rock under different filling schemes.

These findings indicate that the displacement of the surrounding rock near the working area is slightly affected by the filling scheme during the excavation process^38^. This can be attributed to the significantly higher stiffness of the surrounding rock compared to the backfill body, and the main function of backfill body is to improve the stress distribution and restrict the displacement in the surrounding rock.

It is also noticeable that the displacement values of surrounding rock in full non-top filling scheme I are more pronounced than the other three schemes, especially for the corresponding displacement higher than 10 cm when the distance from the working area surpasses 25 m. According to the production experience with relevant literature, a permissible displacement value of 10 cm was selected as the scope of the calculated displacement, to ensure the working areas retain their functionality^39,40^. Therefore, the full non-top filling scheme I can not meet the requirements of displacement control, while the filling every other three scheme IV seems more feasible among the four designed schemes.

## Conclusion

In this study, a numerical simulation method was applied for the comparisons of four backfill schemes. Following conclusions have been reached:

1. Backfill enhances the structural integrity and stability of the surrounding rock, as indicated by the maximum principal stress analysis. The load-bearing capacity of the backfill is limited due to its low stiffness. Simply increasing the backfill volume and strength without consideration on the original capacity of the surrounding rock may potentially result in substantial backfill costs and waste of safety factor margin.
2. The compressive stresses in the artificial roof were maintained lower than the safety threshold of 4.0 MPa. The internal stress values were recorded around 2.0 MPa, lower than the compressive strengths of the backfill.
3. Results of displacement analysis indicate that there is no evident change in the displacement of the upper surrounding rock under different filling schemes, with a range of the 10–12 m range from the bottom of working area in this mining stage. The continuously low displacement values of the backfill body reveal a significant impact of backfilling on reducing the displacement of surrounding rock.
4. The displacement values observed in the upper layers for full non-top filling scheme (I) surpass the scope of the calculated displacement of 10 cm, indicating a higher risk and unsuitability for this scheme. Conversely, the filling every other three scheme (IV) is suggested to replace the current scheme in Shanjin gold mine, due to the superior stability of the surrounding rock. Furthermore, an on-site test regarding the optimized scheme (IV) is then proposed to enhance the future safety and sustainability for the mine.

## Data Availability

The datasets used and/or analyzed during the current study available from the corresponding author on reasonable request.
